# 

**Published:** 1846-09

**Authors:** 


Review of the Proceedings of Last Meeting,.......... 102

Enlargement of the Journal and Library of Dental Science,.......... 106

A New Drill-Stock,.......... 108

Mississippi Valley Association of Dental Surgeons,.......... 108

Virginia Society of Surgeon Dentists,.......... 109

Pennsylvania Association of Surgeon Dentists,.......... 109

Accidents in the Extraction of Teeth,.......... 109

Arkansas Oil-Stone,.......... 110

Filling Teeth,.......... 110

Dental Exostosis,.......... 111

Transposition of Teeth,.......... 111

Opening Address,.......... 112

Ba,timore College of Dental Surgery,.......... 112

To Subscribers,.......... 112

### BIBLIOGRAPHICAL NOTICES.

The Surgical, Mechanical, and Medical Treatment of the Teeth, including Dental Mechanics,.......... 113

Annual Announcement of the Jefferson Medical College of Philadelphia,... 115

Annual Circular of the Massachusetts Medical College,.......... 119

